# Genetic Parameters of Performance and Conformation Traits of 3-Year-Old Warmblood Sport Horses in the Czech Republic

**DOI:** 10.3390/ani12212957

**Published:** 2022-10-27

**Authors:** Alexandra Novotna, Alena Birovas, Hana Vostra-Vydrova, Zdenka Vesela, Lubos Vostry

**Affiliations:** 1 Department of Genetics and Breeding of Farm Animals, Institute of Animal Science, Pratelstvi 815, 10401 Praque, Czech Republic; 2 Department of Ethology and Companion Animal Science, Czech University of Life Sciences Prague, Kamycka 129, 16521 Praque, Czech Republic; 3 Department of Genetics and Breeding, Czech University of Life Sciences Prague, Kamycka 129, 16521 Praque, Czech Republic

**Keywords:** performance test, conformation traits, genetic parameters, animal model, heritability, genetic correlation, horse

## Abstract

**Simple Summary:**

Since 2014, the genetic evaluation of the population of Czech sport horses has been based only on the evaluation of results from jumping competitions. The performance test is an integral part of the testing of young horses in the Czech Republic, as is the linear description of horses. The results from both the linear description and the performance test show low to medium heritability for the observed traits. The genetic parameters obtained in this study indicate that the data obtained from the performance test are suitable for inclusion in the genetic evaluation of sport horses in the Czech Republic, as the breeding values would be refined, especially in young mares and stallions.

**Abstract:**

The aim of this study was to estimate the genetic parameters of a one-day performance test together with the linear type traits of 3-year-old warmblood horses. The study of genetic parameters was based on 5958 tested horses in the period 1998–2021. A total of 22 traits of linear description, three quantitatively measured traits, and one summary mark from the performance test were tested. The model equation included the fixed effect of gender and combination effects of classifier–year of evaluation–place. A single-trait animal model was used for the estimation of heritability and genetic variance, while the two-trait animal model was applied for the estimation of variance and covariance between all traits. The heritability of the overall score of the performance test was 0.25. The range for heritability was between 0.04 and 0.33 for the linear type traits and between 0.46 and 0.57 for the quantitatively measured traits. Genetic correlations were between −0.47 and 0.92. The estimated genetic parameters suggest that the results from the performance test can be incorporated into genetic evaluation in the Czech Republic.

## 1. Introduction

Warmblood horse breeding in the Czech Republic (CZE) primarily concerns jumping performance. Evaluation is based solely on sport competition results, the only currently applicable information for genetic evaluation of Czech sport horses. The genetic merit of the Czech sport horse population has been heavily impacted by imported genetic material [1]. Incorporating the evaluation of performance tests or linear descriptions into the overall genetic evaluation is common in several countries around the world [2,3,4], as it is a simple method of achieving breeding progress. The use of linear description data for genetic evaluation purposes is often welcomed and appreciated by breeders, and breed organizations perceive it as an important service to their breeders [5]. One of the first studies with linear descriptions of conformation and gait traits was carried out for Royal Dutch warmblood horses (KWPN) in 1989 [2].

The current trend of breeding sport horses is a combined selection for conformation and performance and is based primarily on results of the performance test and evaluations of conformation and gait [5]. These attributes are available at a young age and with higher heritability, and together with their correlations with selection traits, they are all important components of modern warmblood breeding programs [6]. Optimal training is crucial for animal welfare. Therefore, an analysis of the changes in routine blood parameters (concentrations of pro-inflammatory cytokines and acute phase proteins) is used to evaluate training progress and general health [7]. The law regulations often forbid any invasive procedures for evaluating blood parameters. Based on the correlations between the blood parameters and changes in body surface temperature parameters, the temperature of body surfaces, measured by infrared thermography (IRL), is used as an alternative procedure to measurements of blood parameters [8]. The IRL may give additional information not only about race training but also about health problems [9]. However, records of these physiological indicators are currently not available in the database of Czech sport horses.

Performance tests are an integral part of horse breeding and are imperative for the breeding of individual breeds. In some countries only station tests are held, and in others both station and one-day field tests are held. The aim of a field test is to test as many animals as possible to obtain information for genetic evaluation and to evaluate the overall quality of the young horses presented in the test. The awarded points, characterizing the evaluated properties, are the basis for the inclusion or, conversely, exclusion of individuals from breeding [10]. 

Heritability of body conformation traits range from 0.09 to 0.28 for KWPN [2], 0.06 to 0.36 for Swedish warmblood horses [11], and 0.07 to 0.18 for Lusitano horses [12]. The traits measured are moderately heritable; for height at withers the authors report a heritability from 0.21 [13] to 0.67 [14], and for cannon bone circumference, a ranking from 0.21 [14] to 0.35 [15]. In performance tests, heritability varies depending on the trait being evaluated. Heritability for gait or movement ranges from 0.17 [12] to 0.36 [3,6]. The coefficient of heritability in sports horses in Hungary for free jumping was 0.29 (jumping style) and 0.52 (jumping ability) [16]. In the Netherlands, the heritability for free jumping is reported in the studbook inspection as ranging from 0.22 (take-off speed, foreleg technique) to 0.37 (scope).

Performance tests are part of the breeding of sports horses in the Czech Republic as well. Mares can only perform a field-performance test at the age of 3, which is called the “basic performance test” (BPT), and has been evaluated since 1994. BPT includes the free jump (100, 110, 120 cm), dressage test, cavalettis in trot with one obstacle, and jumping line. It is assessed on a 10-point scale and marks are awarded for movement and maneuverability (significance coefficient 0.4), innate ability and willingness (significance coefficient 0.4), and submission (significance coefficient 0.2). The overall score is the sum of the partial evaluations multiplied by the significance coefficient. Mares are evaluated (linear description) most often before being entered in the studbook because mares without a linear description are not included in the performance test. Stallions can perform a station test (70 days) or the owner can prepare him for a test similar to that of mares: a one-day field-performance test.

The main goal of this work was to estimate the genetic parameters of the field-performance test of 3-year-old mares and stallions as well as utilize a linear description including heritability and genetic correlations between the evaluated traits. 

## 2. Materials and Methods

### 2.1. Description of Data

The database was obtained from the Central Register of Horses in the CZE for the period 1998–2021. The database included 7418 data entries on horses with a linear description and a total overall score of the performance test from the BPT of mares and from the field-performance test of stallions. A total of 22 linear description traits were evaluated and the system of evaluation was based on a biological scale from one extreme to the other. Classifiers can give marks of 1–9 in increments of 1 point. Three quantitatively measured traits expressed in centimeters and one overall score from the performance test were scored on a scale from 1 (very bad) to 10 (excellent), with increments of 0.1 (Table 1).

A total of 33 breeds were included in the analysis, of which the most numerous breeds were the Czech warmblood (81.7%), the Slovak warmblood (12.9%), the Holstein horse (0.7%), the Oldenburg horse (0.6%), and Hanoverian horse (0.5%). Over a 24-year period, a total of 18 judges evaluated horses aged 3 years, most often between 29 and 46 months. An average of 245 mares and only 4 stallions underwent a performance test annually.

### 2.2. Estimates of (Co)Variance Components, Correlations, and Heritability

To determine the variance components, the database had to be prepared and cleaned of irrelevant and meaningless data. Some horses had repeated records (756 horses); therefore, only the first recorded linear scoring was taken, and the second or third linear scores were deleted. Sires with less than five measured offspring were excluded from the database. The combination effect of classifier–year of evaluation–place had to contain a sufficient number of cases (minimum 10 in the group). If the horses did not have all 26 evaluated traits, they were discarded. The database, suitable for genetic analysis, included data from 5958 horses (5884 mares, 84 stallions).

A preliminary general linear model (PROC GLM; SAS Inst. Inc., Cary, NC, USA) was run on the fixed effects and included the effects of sex and the combination of classifier–year of evaluation–place.

Single-trait and two-trait animal models, including the 22 linear traits, three quantitatively measured traits, and one overall score, were used to estimate the genetic and residual variance, using the average information-restricted maximum likelihood (AI-REML) method. The AIREMLF90 procedure in the BLUPF90 software was used [17]. The animal linear mixed model for the single-trait and two-trait analyses was as follows:

*Y* = 𝑋𝛽 + 𝑍𝑎 + 𝑒 (1)

where *Y* indicates the vector of the 22 linear traits, the three quantitatively measured traits, and one overall score; 𝛽 is the vector of the fixed effects, including sex (2 levels); classifier–year of evaluation–place (231 levels); 𝑎 is the vector of the random animal additive genetic effects; 𝑒 is the vector of the random residual effects; and 𝑋 and 𝑍 are the incidence matrices assigning observations to fixed and random animal effects.

The random effects were assumed to follow normal distributions, with the (co)variance structures as below:

(2) $$\mathrm{Var}\left|\begin{array}{c} a\\ e \end{array}\right|=\left|\begin{array}{c} G⊗A\;\;\;\;0\\ 0\;\;\;\;R⊗I \end{array}\right|;\;G=\left|\begin{array}{c} \sigma_{a1\;}^{2}\;\sigma_{a12}\\ \sigma_{a12}\;\sigma_{a2\;}^{2} \end{array}\right|;\;R=\left|\begin{array}{c} \sigma_{e1\;}^{2}\;\sigma_{e12}\\ \sigma_{e12}\;\sigma_{e2\;}^{2} \end{array}\right|$$

where G is an additive genetic covariance matrix of order 2 × 2; A the additive genetic relationships matrix for p animals; R a residual covariance matrix of order 2 × 2; I is the identity matrix; ⊗ is the Kronecker product operator; σ^2^_a1_, σ_a12_, and σ^2^_a2_ are the additive genetic variance for the two traits and their covariance; and σ^2^_e1_, σ_e12_, and σ^2^_e2_ are the residual (co)variance for the traits.

In order to obtain positive definite variance–covariance matrices, the genetic variance–covariance matrices from the two-trait analysis were modified using the “bending” method [18]. The standard error of the heritability estimates was calculated according to the method of Klei and Tsuruta [19].

(3) $${{\mathrm{SE}}_{h}}^{2}={\left(\frac{a}{p}\right)}^{2}\;\text{×}\;\left\{\frac{\mathrm{Var}\left(a\right)}{a^{2}}+\frac{\mathrm{Var}\left(p\right)}{p^{2}}-2\frac{\mathrm{Cov}\left(a,p\right)}{\mathrm{ap}}\;\right\}$$

A total of 30,281 horses were included in a four-generation pedigree. Phantom groups (fifth generation) were created according to the origin of the founder (breed or breed group). A total of 10 unknown ancestral groups were created in this way: the English thoroughbred (the number of individuals in the group: 1946), Czech origin (407), German origin (1840), Eastern group—Iranian origin (208), Eastern group—Arab origin (173), Eastern group—Berber-Arab origin (132), Nordic group (47), Western group (99), other warmblood origin (357), and unknown breed (1660). Individuals with unknown parents represented 22.7% of the total number of individuals in the pedigree. 

## 3. Results

### 3.1. Description of the Data

The evaluated database showed (Table 2) that the average of the linear traits ranged from 4.77 (back shape) to 6.77 (stride length of trot), and the average of the quantitatively measured traits were in the range of 20.65 (cannon bone circumference) and 190.54 cm (heart girth). The average overall score was 7.59, with a standard deviation of 0.53. The lowest overall score awarded in the performance test was 4.38 and the highest 9.60. Most of the linear traits had a mean and mode close to 5; that is, the mean point of the linear scale. The highest average marks were recorded for stride length of walk and trot (6.72; 6.77) and type (6.48), and the lowest for back shape (4.77), hind hoof (4.91), and front hoof (4.92). Higher values are preferred especially for movement and overall score; for other linear traits, the optimum is usually around 5 points, but it always depends on the described traits. 

### 3.2. Estimates of the (Co)Variance Components

The results of the preliminary analysis carried out with the GLM method showed that the combination of classifier–year of evaluation–place effects was statistically significant (*p* < 0.001) for all observed traits. The gender effect was statistically significant only for certain traits: croup length, croup slope, height at withers, heart girth, cannon bone circumference, and overall score. Residual variance, expressed as the root mean square error, ranged from 0.32 (hind hoof) to 6.15 (heart girth).

For the traits of linear description (Table 3), the highest genetic variance (σ²a) was found in nobleness (0.332), body width (0.139), and croup shape (0.136). The standard error (SE) of the genetic variance ranged from 0.002 (hind hoof) to 0.036 (nobleness). The highest phenotypic variance (σ²_p_) was found for type (0.713), nobleness (0.681), shoulder (0.670), and frame (0.670). The standard error of the phenotypic variance ranged from 0.003 (hind hoof) to 0.030 (nobleness). Among the quantitatively measured traits, the highest genetic variance was found in heart girth (19.178), followed by height at withers (7.951) and cannon bone circumference (0.345); their standard error ranged from 0.027 to 1.776. For overall score, the genetic variance was 0.054, with a standard error of 0.007, and for phenotypic variance it was 0.159, with standard error of 0.006.

The highest heritability was found for the quantitatively measured traits (Table 3). The traits of linear description were low to moderate, ranging from h^2^= 0.04 (hind pastern) to h^2^= 0.33 (nobleness). The overall score was h^2^ = 0.25. The standard error of heritability ranged from SE_h_^2^ = 0.017 to SE_h_^2^ = 0.027.

### 3.3. Genetic and Phenotypic Correlations

Genetic correlations were obtained with two-trait analysis and ranged from −0.47 to 0.92 (Table 4). There were positive, high genetic correlations between the upper line and legs. On the contrary, negative, medium-high genetic correlations were found for croup slope and croup length, length of withers and neck position, and stride length of walk and hind pastern. All quantitatively measured traits were also highly correlated. Overall score was the most correlated with gait and frame. Other traits related to the overall frame of the horse and its top line (neck length, neck position, croup length, croup shape, and body width) were correlated with the overall score in the range of 0.18 to 0.23. Other traits did not have a significant genetic correlation with the overall score.

Phenotypic correlations show that they usually have significantly lower values than genetic correlations, often fluctuating around zero. Medium-high phenotypic correlations were found only between the quantitatively measured traits and between the stride length of the walk and trot.

## 4. Discussion

Heritability estimates differ in dependence on the group of conformation traits (Table 3). Heritability for overall impression was reported at both low (Ref. [12]; h^2^ = 0.14) and moderate levels (Ref. [16]; h^2^ = 0.43). In another study of the Czech sport horse population [6], the heritability for the type was reported as 0.24. The different values of the heritability for the type traits in Czech studies is caused by a very pre-selected group of animals. The population of horses is more uniform by type because all animals should be used in sport. A degree of typicality can also express the properties of the utility group (jumping type). Heritability estimates for the upper line of horse—neck, withers, back, loins, and croup—were also still in line with other studies [2,13,15], while the results of the heritability estimates for legs, hoof, and pasterns differ. For the Dutch warmblood horses, the values ranged from 0.14 to 0.23 [2]; for Czech cold-blooded horses, it ranged from 0.11 to 0.37 [20]; for the Andalusian horse, it was 0.29 [15]; and for the Italian heavy draught horse, 0.06 [21]. 

In other sports horse breeds, higher heritability for gait has been recorded than in this study. Swedish warmbloods, reported from the RHQT test, showed 0.29 (walk) and 0.40 (trot) [10]; Dutch warmbloods, from the FSI test, showed 0.35 (walk) and 0.50 (trot) [22]; Belgian warmblood horses showed 0.33 to 0.52 [23]; and German warmblood horses showed 0.38 for walk and 0.49 for trot, based on the mares’ performance test [24]. These authors reported significantly higher heritability for gait because their studies were based on the evaluation of gait in the performance test. In the performance test, gait is often evaluated as part of a performance test. Individual sub-traits of gait, such as correctness, stride, elasticity, impulsion, and suppleness, are often recorded, which provides a more detailed description of the individual gait and does not result in loss of variability.

The heritability of the quantitatively measured traits were moderately high, ranging from 0.46 to 0.57, which is mostly consistent with other works [15,19,20,25]. Slightly higher heritability for height at withers (0.67) was recorded in the Dutch warmblood horse population [26], in Icelandic horses (Ref. [4]; h^2^ = 0.67), and in Swedish warmblood horses (Ref. [10]; h^2^ = 0.84). On the contrary, slightly lower heritability for cannon bone circumference (0.35) was estimated in Dutch warmblood horses [26]. 

The overall score is an overall evaluation of the performance test. It includes evaluations of movement and manageability, innate abilities, and willingness and submission. In the Czech Republic, the partial traits of the performance test are recorded only in paper form, and these partial traits are no longer stored in the national database because it is not required by breeding organizations. Therefore, in this study, only the overall score of the performance test was evaluated—the only one recorded in the database. In most foreign studbooks, partial traits from performance tests are thoroughly recorded and evaluated. The overall evaluation of the performance test, summarized in one mark, is used more for the overall evaluation of the walk, trot, or gallop, or for the overall evaluation of the free jump. For example, in the old Kladruber horse, overall impression is evaluated as a separate trait, with a heritability of 0.33 in the performance test [27]. In the Hungarian Sport Horse population, overall impression is included in the Sport Horse self-performance test as movement analysis, with an estimated heritability of 0.33 [16]. In this study, the estimated heritability for the overall score is 0.25. In some works [10,15,21], overall evaluation of body conformation is most often included under the overall score. For Lusitano horses, it is used for the studbook registration final score, which is the sum of the eight partial scores for morphology and gait, obtained by weighing the partial scores [12]. For German warmblood riding horses, the total score is also calculated as the mean of the total conformation (weighted average of the six individual scores) and the individual scores for type correctness of gait, impetus and elasticity, walk at hand, and development [28].

The highest genetic correlations (Table 4) were found between frame within back length and length of loins, and also between front and hind pastern. The high correlation between frame and back and loins is understandable because the given traits overlap to a certain extent. For sport horses, longer lines are required from a long neck, a long topline, including the back, and a long sloping shoulder. Body width is moderately positively correlated with croup shape and height at withers. Walk is moderately correlated with trot. The walk was negatively correlated with hind pastern (−0.42) because a slope pastern negatively affects the stride length of the walk. Other negative correlations were found between nobleness and height at withers, heart girth, and cannon bone circumference. Nobleness is usually expressed by gentle and harmonious body shapes and the expression of the head and neck, while the entire structure of the body should be in harmonious proportions. Large and rapidly growing horses would be more predisposed to unsoundness and bone-development disorders [11]. The estimated correlation for the overall score in this study (Table 4) is in accordance with a study conducted with Lusitano horses, where the authors found that the genetic correlation between the gait and final score was 0.678 [12]. Traits related to the overall frame of the horse and its top line (neck length, neck position, croup length, body width, and croup shape) were correlated in the range of 0.18 to 0.22. The overall evaluation of the horse in the performance test is therefore also influenced by its type and stride length in walk and trot. A horse’s forelimbs naturally carry most of the body’s weight, and the role of head–neck position in horses for movements at walk and trot and back movements has been demonstrated in other studies [29,30].

The genetic evaluation of the conformation traits in combination with performance traits certainly brings new knowledge to the overall view of the genetic studies of horse breeding at the national and international level. In general, it is difficult to compare the evaluation of body conformation across different breeds because each breed has a different breeding goal and method of evaluation [14]. Many studies are based on the evaluation of a linear description, where a different number of traits are individually evaluated according to a biological scale, such as the Dutch warmblood horse [2], Swedish warmblood horse [11], Haflinger horse [31], and Old Kladruber horse [32,33]. The linear description can also be evaluated simultaneously with the performance test [11]. The simultaneous evaluation of body conformation traits and performance traits brings an advantage in the form of genetic correlations between individual traits [12]. Multiple-trait models, including both body conformation traits and performance traits, can increase the accuracy of the EBV for these traits. In a breeding program, pre-selection for conformation traits before a performance test can lead to a correlated response to the performance traits [23].

Marker-assisted selection is a novel technique in horse breeding; for example, in Thoroughbreds, it is well established that polymorphisms in the myostatin gene (MSTN) is a major quantitative trait locus, and is the single most influential gene on distance aptitude [34]. A genome-wide association study found an SNP on chromosome 18 (ECA18) to be the most significant SNP on the Illumina EquineSNP50 genotyping array when using best race distance as a quantitative phenotype [35]. The identification of SNP markers linked to the myostatin loci that are associated with athletic ability in the Thoroughbred horse represents the first gene influencing athletic ability identified in horses [36]. Myostatin in the Thoroughbred has become known as the “speed gene” and is now widely used as a predictor of optimum race distance in Thoroughbreds and mate selection for breeding for specific racing traits [37].

In the Czech Republic, sports horses are tested from an early age. There are exhibitions of mares with foals, test breeding facilities for young stallions, a station test for 3-year-old stallions, a field test for 3-year-old mares and stallions, special breeding competitions (show jumping, dressage, eventing) for horses from 4 to 6 years old, and equestrian competitions. It seems that many different tests could be used to evaluate their genetics, but this is not the case. To date, the only possible genetic evaluation is an evaluation based on the database of the show jumping results, as this database is not subject to significant pre-selection, and for each horse the results from multiple competitions are mostly known [38]. In other cases, there are not enough data for genetic evaluation (the breeder is not obliged to participate in any test), and often the results are recorded only in paper form without being stored in databases. Another problem lies in the exchange of data at the national level, namely, between breeding associations, the Central Register of Horses, and the Czech Equestrian Federation, because not every organization has the same method of storing data in databases.

## 5. Conclusions

The genetic parameters for the conformation and performance traits obtained in this study are useful for selection programs of the Czech sport horse population. The favorable genetic correlations indicate that selection for one trait can evoke a favorable indirect genetic response in another trait. The combined use of the genetic evaluation of show jumping with the genetic evaluation of the performance test will facilitate and accelerate selection progress, especially of the broodmare. This would be desirable both for the selection of potential warmblood broodmares and also for a reduction in the generation interval. The analysis also shows that, in addition to the overall score, it would be appropriate to record all sub-assessed traits in the performance test. Summarizing into a single mark is not appropriate because by averaging the traits one will lose too much of the information; thus, the genetic evaluation will lose its reliability.

## Figures and Tables

**Table 1:** Description of the conformation and performance traits used for the entry of horses into a studbook.

Trait	Scale 1	Scale 9
**Conformation traits** Type (Ty)	Markedly atypical	Markedly typical
Frame (Fr)	Very short frame	Very long frame
Nobleness (Nob)	Serious irregularities in harmony	Very noble to fine
Neck length (NLe)	Very short	Very long
Neck position (NPo)	Very low-set	Very high-set
Length of withers (LeW)	Very short	Very long
Back length (BLe)	Very short	Very long
Back shape (BSh)	Pronounced sway back	Pronounced roach back
Length of loins (LeL)	Very short	Very long
Shape of loins(ShL)	Sunken loins	Vaulted loins
Croup length (Cle)	Very short	Very long
Croup slope (CSl)	Flat	Markedly steep
Shoulder (Sho)	Upright and short	Sloping and very long
Front pastern (FP)	Upright pastern	Very sloping pastern
Front hoof (FH)	Very flat	Flexural contraction
Formation of hind legs (FHL)	Open angle of hocks	Sickle hocks
Hind pastern (HP)	Upright pastern	Very sloping pastern
Hind hoof (HH)	Flat	Flexural contraction
Body width (BW)	Very narrow	Markedly wide
Croup shape (CSh)	Rafter and narrow	Split croup
Stride length of walk (SLW)	Markedly short	Very long, flexible
Stride length of trot (SLT)	Markedly short	Very long
Height at withers (HW)	In centimeters	
Heart girth (HG)	In centimeters	
Cannon bone circumference (CBC)	In centimeters	
Performance trait Overall score of performance test (OS)	Scale 1–10	0.1 point

**Table 2:** Descriptive statistics of the 26 evaluated traits.

Trait	Mean	SD	Range	Mode
**Conformation traits** Type (Ty)	6.48	1.04	2–9	7
Frame (Fr)	5.55	1.00	1–9	5
Nobleness (Nob)	6.03	1.16	3–9	6
Neck length (NLe)	5.43	0.89	3–8	5
Neck position (NPo)	4.93	0.78	2–9	5
Length of withers (LeW)	5.77	0.85	3–9	6
Back length (BLe)	5.40	0.78	3–8	5
Back shape (BSh)	4.77	0.57	1–8	5
Length of loins (LeL)	5.59	0.67	3–8	6
Shape of loins (ShL)	5.01	0.64	2–8	5
Croup length (Cle)	5.11	0.84	2–8	5
Croup slope (CSl)	5.57	0.81	2–8	5
Shoulder (Sho)	5.40	1.04	2–9	5
Front pastern (FP)	5.00	0.58	2–9	5
Front hoof (FH)	4.92	0.56	2–8	5
Formation of hind legs (FHL)	5.07	0.79	2–8	5
Hind pastern (HP)	4.94	0.50	2–8	5
Hind hoof (HH)	4.91	0.37	3–8	5
Body width (BW)	5.31	0.92	2–8	5
Croup shape (CSh)	5.37	0.89	2–8	5
Stride length of walk (SLW)	6.72	0.92	3–9	7
Stride length of trot (SLT)	6.77	0.95	3–9	7
Height at withers (HW)	164.05	3.93	129.0–182.0	165.0
Heart girth (HG)	190.04	6.61	119.0–228.0	190.0
Cannon bone circumference (CBC)	20.65	0.82	15.9 ± 26.5	21.0
**Performance trait** Overall score of performance test (OS)	7.59	0.53	4.38–9.60	7.6

**Table 3:** Estimated genetic (σ²a) and phenotypic (σ²p) variance and heritability (h^2^) obtained from the single-trait analysis.

Trait	σ²a (SE)	σ²p (SE)	h^2^ (SE)
**Conformation traits** Type (Ty)	0.087 (0.019)	0.713 (0.021)	0.11 (0.021)
Frame (Fr)	0.076 (0.018)	0.670 (0.020)	0.10 (0.021)
Nobleness (Nob)	0.332 (0.036)	0.681 (0.030)	0.33 (0.025)
Neck length (NLe)	0.054 (0.013)	0.514 (0.015)	0.10 (0.021)
Neck position (NPo)	0.052 (0.012)	0.445 (0.013)	0.10 (0.020)
Length of withers (LeW)	0.037 (0.011)	0.502 (0.014)	0.07 (0.019)
Back length (BLe)	0.051 (0.011)	0.393 (0.012)	0.11 (0.021)
Back shape (BSh)	0.022 (0.006)	0.260 (0.007)	0.08 (0.020)
Length of loins (LeL)	0.056 (0.010)	0.321 (0.010)	0.15 (0.023)
Shape of loins (ShL)	0.024 (0.007)	0.342 (0.009)	0.06 (0.016)
Croup length (Cle)	0.036 (0.009)	0.438 (0.012)	0.08 (0.018)
Croup slope (CSl)	0.069 (0.013)	0.428 (0.014)	0.14 (0.023)
Shoulder (Sho)	0.027 (0.012)	0.670 (0.017)	0.04 (0.017)
Front pastern (FP)	0.030 (0.008)	0.292 (0.009)	0.09 (0.021)
Front hoof (FH)	0.036 (0.007)	0.249 (0.008)	0.13 (0.022)
Formation of hind legs (FHL)	0.079 (0.015)	0.484 (0.016)	0.14 (0.023)
Hind pastern (HP)	0.009 (0.004)	0.229 (0.006)	0.04 (0.017)
Hind hoof (HH)	0.007 (0.002)	0.098 (0.003)	0.07 (0.018)
Body width (BW)	0.139 (0.021)	0.557 (0.020)	0.20 (0.024)
Croup shape (CSh)	0.136 (0.019)	0.494 (0.018)	0.22 (0.024)
Stride length of walk (SLW)	0.069 (0.014)	0.507 (0.015)	0.12 (0.021)
Stride length of trot (SLT)	0.102 (0.019)	0.562 (0.019)	0.15 (0.024)
Height at withers (HW)	7.951 (0.636)	6.698 (0.459)	0.54 (0.026)
Heart girth (HG)	19.178 (1.776)	22.104 (1.338)	0.46 (0.027)
Cannon bone circumference (CBC)	0.345 (0.027)	0.264 (0.019)	0.57 (0.026)
Performance trait Overall score of performance test (OS)	0.054 (0.007)	0.159 (0.006)	0.25 (0.025)

**Table 4:** Estimates of the genetic (above the diagonal) and phenotypic (below the diagonal) correlations, as computed by the two-trait analysis.

Trait	Ty	Fr	Nob	NLe	NPo	LeW	BLe	BSh	LeL	ShL	Cle	CSl	Sho	FP	FH	FHL	HP	HH	BW	CSh	SLW	SLT	HW	HG	CBC	OS
Ty	−0.02	0.39	0.39	0.28	0.33	−0.22	−0.03	−0.09	−0.13	0.33	−0.17	0.10	0.20	0.50	−0.12	0.09	0.28	0.49	0.41	0.21	0.52	0.50	0.49		0.40	0.52
Fr	0.07		−0.36	0.08	0.06	−0.19	0.94	0.11	0.72	−0.28	0.30	−0.18	0.07	−0.36	−0.13	0.08	−0.09	0.13	0.36	0.18	−0.27	0.02	−0.04	0.16	0.30	−0.11
Nob	0.25	0.03		0.49	0.09	0.25	−0.22	−0.14	−0.20	−0.28	0.10	0.20	0.26	−0.12	0.30	−0.17	0.18	0.23	−0.26	−0.27	0.29	0.16	−0.38	−0.47	−0.62	0.15
NLe	0.18	0.10	0.11		0.60	−0.06	0.28	−0.01	0.11	−0.36	0.00	−0.23	−0.07	0.09	0.18	0.03	0.17	0.15	−0.23	−0.14	0.09	0.16	0.26	0.12	−0.14	0.18
NPo	0.17	0.03	0.10	0.08		−0.43	0.09	−0.41	0.26	−0.17	0.07	−0.35	0.04	−0.09	0.19	0.32	−0.04	0.25	0.12	0.36	−0.23	0.13	0.28	0.29	0.11	0.23
LeW	−0.02	0.06	−0.01	0.05	−0.08		−0.20	0.36	0.05	0.33	−0.05	0.36	0.05	0.29	0.09	−0.19	0.01	−0.23	−0.11	−0.07	0.25	0.27	0.28	−0.12	0.05	0.12
BLe	−0.01	0.30	−0.04	0.05	0.02	−0.02		−0.24	0.92	−0.22	0.09	−0.20	−0.14	−0.27	−0.16	0.24	0.06	0.27	−0.12	0.01	−0.36	−0.22	−0.10	−0.20	0.08	−0.05
BSh	0.03	−0.06	0.03	0.01	−0.03	0.03	−0.03		0.32	0.35	−0.12	0.34	0.19	0.17	−0.22	0.07	0.18	−0.17	−0.29	−0.44	0.39	0.14	0.28	−0.10	0.23	−0.14
LeL	−0.08	0.23	−0.04	0.02	−0.05	0.09	0.37	−0.01		0.08	−0.11	−0.17	0.05	0.07	0.02	0.22	0.12	0.24	0.02	−0.08	−0.11	−0.17	0.24	0.01	0.26	−0.11
ShL	0.02	0.03	0.00	0.00	−0.03	−0.01	0.00	0.19	0.03		0.42	−0.13	−0.20	0.21	−0.24	−0.02	0.16	−0.13	−0.13	0.18	0.31	0.10	0.30	0.30	0.28	−0.13
Cle	0.20	0.07	0.04	0.15	0.13	−0.04	0.07	0.02	0.02	0.00		−0.55	−0.05	−0.13	0.01	0.09	0.00	0.23	0.27	0.58	−0.01	0.12	−0.01	0.38	0.27	0.21
CSl	−0.07	−0.03	−0.04	−0.02	−0.04	0.08	−0.05	−0.02	−0.01	0.04	−0.12		0.33	−0.18	−0.02	−0.11	0.11	−0.15	−0.09	−0.33	0.16	−0.03	−0.04	−0.27	−0.12	−0.12
Sho	0.15	0.05	0.06	0.07	0.08	0.03	0.02	−0.04	0.00	−0.03	0.17	0.01		0.03	0.25	0.30	0.14	0.08	−0.15	0.00	0.17	0.19	−0.22	0.08	−0.07	0.11
FP	0.00	0.02	0.00	0.01	0.00	−0.02	0.03	0.05	0.00	−0.01	0.01	0.01	−0.03		0.34	−0.27	0.82	0.34	0.07	0.03	−0.12	0.11	0.32	0.25	0.13	−0.18
FH	−0.04	0.01	0.02	0.00	0.02	−0.02	0.01	0.04	−0.01	0.01	0.01	0.00	−0.03	0.17		−0.13	0.02	0.62	0.09	−0.03	0.08	−0.01	0.17	−0.04	0.03	0.17
FHL	−0.02	0.02	−0.02	0.00	−0.03	0.04	0.02	−0.01	−0.01	−0.03	−0.01	0.03	0.04	0.07	0.04		−0.33	−0.05	−0.14	−0.03	0.04	0.00	0.15	0.04	0.18	−0.02
HP	0.05	0.01	−0.01	0.03	−0.01	0.02	−0.02	0.04	−0.01	0.01	0.00	0.01	0.01	0.19	0.08	0.08		0.22	0.15	0.04	−0.42	−0.13	0.01	0.22	0.22	0.05
HH	0.02	−0.02	−0.06	−0.01	−0.01	0.00	−0.02	0.00	−0.02	0.02	−0.01	0.02	0.01	0.02	0.18	0.02	0.20		−0.08	0.02	−0.02	0.22	−0.05	−0.06	−0.02	0.22
BW	0.16	0.04	−0.04	0.13	0.15	−0.08	0.07	−0.01	0.02	0.00	0.22	−0.10	0.10	−0.02	−0.03	−0.01	−0.01	0.03		0.65	0.08	0.08	0.02	0.63	0.43	0.20
CSh	0.20	0.01	0.12	0.11	0.14	−0.08	−0.03	−0.03	−0.07	−0.05	0.22	−0.04	0.12	0.03	0.02	−0.01	0.02	0.01	0.34		0.08	0.12	0.05	0.42	0.28	0.20
SLW	0.19	0.04	0.02	0.09	0.08	0.07	0.04	−0.01	−0.01	−0.02	0.09	−0.03	0.10	−0.02	−0.03	0.00	0.01	0.01	0.05	0.09		0.48	0.11	−0.04	0.00	0.49
SLT	0.17	0.03	0.01	0.07	0.10	0.04	0.03	−0.01	0.00	0.01	0.00	−0.03	0.09	−0.05	0.00	0.04	0.02	0.02	0.08	0.11	0.47		0.23	0.16	0.09	0.58
HW	0.08	0.01	−0.12	−0.01	−0.06	0.09	0.09	0.07	−0.01	−0.02	0.05	0.03	0.05	−0.03	−0.06	0.04	0.04	0.08	0.06	−0.08	0.10	0.12		0.71	0.72	0.07
HG	0.11	0.02	−0.11	0.02	0.08	−0.02	0.09	0.02	0.00	−0.04	0.13	−0.04	0.05	−0.01	−0.02	0.02	0.02	0.04	0.29	0.15	0.06	0.05	0.34		0.77	0.11
CBC	0.03	−0.01	−0.13	0.04	0.01	0.07	0.09	0.00	0.01	−0.01	0.05	−0.01	0.05	−0.02	−0.01	0.01	0.00	0.10	0.12	0.00	0.10	0.10	0.38	0.22		0.10
OS	0.07	0.01	0.04	0.04	0.00	0.01	−0.01	0.03	−0.03	0.01	0.02	0.01	0.04	−0.01	−0.04	0.01	−0.01	−0.03	−0.01	0.00	0.13	0.12	0.05	0.03	−0.03	

## Data Availability

Restrictions apply to the availability of these data. Data was obtained from the Central Register of Horses in the CZE and are available from the authors with the permission of the Central Register of Horses in the CZE.
